# Prevalence of anemia and its associated factors among children aged 6–59 months in the Lao People’s Democratic Republic: A multilevel analysis

**DOI:** 10.1371/journal.pone.0248969

**Published:** 2021-03-25

**Authors:** Sengtavanh Keokenchanh, Sengchanh Kounnavong, Kaoru Midorikawa, Wakaha Ikeda, Akemi Morita, Takumi Kitajima, Shigeru Sokejima

**Affiliations:** 1 Department of Public Health and Occupational Medicine, Mie University Graduate School of Medicine, Tsu-shi, Mie, Japan; 2 Foreign Relation Division, Cabinet of the Ministry of Health, Sisattanack District, Vientiane Capital, Lao PDR; 3 Lao Tropical and Public Health Institute, Ministry of Health, Sisattanack district, Vientiane Capital, Lao PDR; 4 Faculty of Child Education, Suzuka University, Suzuka, Mie, Japan; 5 Epidemiology Centre for Disease Control and Prevention, Mie University Hospital, Tsu-shi, Mie, Japan; School of Public Health, University of São Paulo, BRAZIL

## Abstract

Anemia is a major public health concern among children aged <5 years in the Lao People’s Democratic Republic. Thus far, no study has determined the factors associated with anemia among children aged <5 years in the Lao People’s Democratic Republic using a nationwide representative sample. Therefore, this study aimed to evaluate the prevalence of anemia and its associated factors with multilevel variations among children aged 6–59 months. This quantitative, cross-sectional study used a nationally representative sample from the Lao Social Indicator Survey II, 2017. Children aged 6–59 months tested for anemia were included in this study through multistage sampling approaches. Anemia was defined as a hemoglobin level of <11.0 g/dL. Multilevel binary logistic regression analyses were used to determine the adjusted effect of the factors associated with anemia. Among the 5,087 children included, the overall prevalence of anemia was 43.0%. Three factors were associated with higher odds of developing anemia—male sex (adjusted odds ratio, 1.16; 95% confidence interval, 1.01–1.34), underweight (adjusted odds ratio, 1.30; 95% confidence interval, 1.09–1.55), and residence in central provinces (adjusted odds ratio, 1.59; 95% confidence interval, 1.30–1.95) and southern provinces (adjusted odds ratio, 1.42; 95% confidence interval, 1.11–1.81). However, the other three factors—age, educational level of the household head, and Hmong-Mien ethnicity—were inversely associated with anemia. To resolve the problem regarding the severity of the anemia among children aged <5 years in the Lao People’s Democratic Republic. Our findings highlight the need for designing an effective approach to address each factor associated with childhood anemia. Interventions should focus on the prevention of childhood anemia, which is considered a major priority of public health intervention in the Lao People’s Democratic Republic.

## Introduction

Anemia (low blood hemoglobin [Hb] levels) is a global public health problem affecting low-, middle-, and high-income countries that has major human health consequences and an adverse impact on social and economic development [1]. In 2011, the global prevalence of anemia in children aged <5 years was 42.6%, translating to approximately 273 million children, and was dominant in Africa and South Asia [1, 2]. According to the World Health Organization (WHO), anemia at the age of 6–59 months is defined as Hb levels <11.0 g/dL [3]. The most common causes of anemia among preschool children in developing countries are nutritional disorders and infections [4]. Various previous studies conducted in different settings have found many household factors associated with an increased risk of developing anemia among children aged <5 years, including children from poor families [5–7], household food insecurity [8], living in urban areas [6], crowded conditions and multiple siblings [7], low maternal education level [6, 7], anemic mothers [8–11], and female household heads [8]. Among the various factors male sex [12], age of 6–23 months [5, 6, 8], and malaria [9] are associated with a higher risk of anemia in children. Moreover, children with a poor nutritional status, including wasting, stunting, and underweight [5, 8, 12], and those having inappropriate complementary food introduction, are more likely to be anemic than their counterparts [7].

Similar to many developing countries, anemia is a major public health problem among children aged <5 years in the Lao People’s Democratic Republic (Lao PDR), where the prevalence of anemia was slightly increased from 40.9% to 42.0% between 2006 and 2011 [1, 13]. However, the prevalence continued be high hereafter; hence, anemia was classified as a condition of severe public health significance based on the WHO classification [3]. The causes of anemia in the Lao PDR are often unknown [13]; however, the national nutrition survey conducted in 2006 reported that anemia in 25%–30% children aged 6–23 months is caused by iron deficiency [14]. Asymptomatic malaria has been increasingly associated with anemia among children in the Lao PDR [15]. However, limited studies have assessed anemia and its associated factors among preschool children in the Lao PDR thus far. Furthermore, these studies have been conducted a limited area [14], and no study has identified factors associated with childhood anemia by controlling for the variations at the individual, household, and community levels. Thus, a better understanding of the determinants of anemia among children aged <5 years using a large sample is needed to improve policy design and effective interventions for addressing anemia among children in the Lao PDR. Therefore, this study aimed to assess the prevalence of anemia and its associated factors with multilevel variations among children aged 6–59 months in the Lao PDR.

## Methods

### Data source and sampling

This study used the Lao Social Indicator Survey (LSIS) II, 2017 dataset from the Lao Statistics Bureau, Ministry of Planning, and Investment in collaboration with the Ministry of Health and Ministry of Education and Sport. This cross-sectional survey provides national figures on social indicators. It combined the Multiple Indicator Cluster Survey (MICS) and the Demographic and Health Survey modules to maximize government resources, which aim to provide up-to-date information with the selection of data on key social development indicators to support monitoring of the Sustainable Development Goals.

Details of the questionnaires, procedures, and methodology used in this survey can be found elsewhere [16]. LSIS II, 2017 was conducted using a multi-stage cluster sampling technique for the selection of the survey sample. The 18 provinces of the Lao PDR were identified as sampling strata. The urban areas and rural areas with and without roads within each province were identified as the main sampling strata, and the sample of households was selected in two stage. The primary sampling units selected during the first stage were villages. A listing of households was conducted in each sample village, and a sample of households was selected at the second stage. The sample included a total of 1,170 villages (373 urban enumeration areas [EAs], 687 rural with road EAs, and 110 rural without road EAs) that were randomly selected using systematic probability proportional to size at the first stage, which were considered EAs, and a fixed number of 20 households were selected from each EA using random systematic selection procedures during the second stage, which resulted in 23,400 households. Due to insufficient budget availability and the time required to complete each cluster, Hb testing was performed among children aged 6–59 months in half of the selected households from the general survey (S1 Fig). A blood sample was drawn into a microcuvette, and Hb analysis was performed onsite with a battery-operated HemoCue analyzer for estimating the Hb level in grams per deciliter blood. The results of Hb testing were shared with the respondents. In cases of severe anemia (Hb level <7.0 g/dL), a letter for referral to the nearest health facility was issued. The LSIS II data were collected based on the MICS6 model questionnaire by qualified and trained interviewers.

### Study variables

The outcome of the present study was anemia, defined as Hb level <11.0 g/dL based on the WHO classification [3]. The independent variables were mostly based on the reviewed literature, which included individual-, household-, and community-level factors.

Individual-level factors included the sex of children, age in months (6–11, 12–23, 24–35, 36–47, and 48–59 months), health insurance status, and health status in the last 2 weeks before the survey, including diarrhea, fever, and illness with a cough. The anthropometric indicators weight-for-age (WAZ), height-for-age (HAZ), and weight-for-height (WHZ) were used to determine nutritional status, which included underweight, stunting, and wasting. Children with a WAZ Z-score of <-2 standard deviation (SD) are considered underweight [17]. Stunting, a phenomenon in which a child is too short for his or her age, is defined as a HAZ Z-score of <-2SD. Wasting, a phenomenon in which a child who is too thin for his or her height, is defined as a WHZ Z-score of <-2SD [18]. The reference population is based on the WHO child growth standards [19].

Household-level factors included age (≤24, 25–34, 35–44, and >44 years), sex of the household head, education level of the household head and mother (none, primary, secondary, and post-secondary level), and ethnicities (Lao–Tai, Mon–Khmer, Hmong–Mien, Chinese–Tibetan, and others). In addition, the household wealth index was determined using the scores derived from the principal component analysis of almost all household assets, and utility services. This variable was already included in the dataset as five quintiles ranked from the poorest to the richest. Moreover, the size of the family was based on the number of household members (<4, 4–6, 7–9, and >9 people). The main sources of drinking water were categorized as improved (piped water, tube well/borehole, protected well, protected spring, rain water collection, tanker truck, and bottled water) and unimproved (unprotected well, unprotected spring, surface water, and others) sources. The type of toilet facilities were categorized as improved (flush to the sewer system, septic tank, pit latrine, ventilated improved pit latrine, pit latrine with slab, and composting toilet) and unimproved (flush to open drain, pit latrine without a slab, hanging toilet, open defecation, and others) toilets, and the availability of mosquito bed nets were also included.

Community-level factors, including the area of residence, were categorized based on sampling stratification as urban areas and rural areas with and without roads, and the regions of residence were categorized based on three geographic regions of the Lao PDR: northern (Phongsaly, Luang Namtha, Oudomxay, Bokeo, Luang Prabang, Huaphanh, and Xayabury), central (Vientiane capital, Xiengkhuang, Xaysomboune, Vientiane province, Borikhamxay, Khammuane, and Savannakhet), and southern (Saravane, Sekong, Champasack, and Attapeu) provinces.

### Statistical analysis

Data analysis was performed using SPSS software, version 25 (IBM Corp., Armonk, NY). Children’s sampling weights were provided in the dataset and included in all analyses to adjust for the effects of the stratification and cluster sampling approaches. Descriptive statistics were used to summarize the anemia status according to each independent variable. Pearson’s chi-squared test was used to determine the factors associated with anemia among children. To provide valid estimates of exposure effects, only variables with a p-value of <0.20 in the bivariate analysis were considered for entry into multivariate analyses [20] after checking for multicollinearity by computing the variance inflation factors. Only variables with variance inflation factors of <2 were included. The multivariate analyses were computed using a multilevel binary logistic regression model with a random intercept at the community and household levels. The results are reported as the crude odds ratio (OR) and adjusted odds ratio (AOR) with 95% confidence intervals (CI). All variables with p-values <0.05 were considered statistically significant for all tests.

### Ethical consideration

The survey protocol was approved by the Lao Statistics Bureau, Ministry of Planning and Investment. Moreover, for the protection of using the secondary data in this study, the research proposal was reviewed and provided ethical approval (ID: 2020.33.NW) by the National Ethics Committee for Health Research, Ministry of Health of the Lao PDR. Interviewers explained the objective of the survey to parents/guardians, and informed verbal consent was obtained from parents/guardians before data collection. The interviewers drew a cycle on the questionnaire if permission was given.

## Results

### Characteristics of the population study

A total of 5,087 children aged 6–59 months (male 51.7% and female 48.3%) were included in this study. Approximately 24.5% children were aged 36–47 months, and most of them (85.9%) were uninsured (Table 1). Most children had a male household head (90.3%), belonged to the Lao-Tai ethnicity (55.4%), lived in rural areas with road areas (62.3%), and resided in central provinces (47.0%). However, approximately 26.2% children lived in the poorest households, with more than nine household members (8.7%), and had an unimproved source of drinking water (17.7%; Table 2).

**Table 1:** Bivariate analysis of childhood anemia according to individual-level factors.

Variables	Total weighted (%)	Non-Anemia, n (%)	Anemia, n (%)	p-value
Sex				**0.004**
Female	2458 (48.3)	1451 (59.0)	1007 (41.0)	
Male	2629 (51.7)	1448 (55.1)	1181 (44.9)	
Age				**<0.001**
6–11 months	544 (10.7)	149 (27.4)	395 (72.6)	
12–23 months	1067 (21.0)	450 (42.2)	617 (57.8)	
24–35 months	1140 (22.4)	714 62.6)	426 (37.4)	
36–47 months	1247 (24.5)	815 (65.4)	432 (34.6)	
48–59 months	1089 (21.4)	771 (70.8)	318 (29.2)	
Health insurance				0.41
No	4372 (85.9)	2481 (56.7)	1891 (43.3)	
Yes	716 (14.1)	418 (58.4)	298 (41.6)	
Underweight				0.14
No	3956 (77.8)	2275 (57.5)	1681 (42.5)	
Yes	1131 (22.2)	623 (55.1)	508 (44.9)	
Stunting				0.72
No	3268 (64.3)	1868 (57.2)	1400 (42.8)	
Yes	1818 (35.7)	1030 (56.7)	788 (43.3)	
Wasting				0.94
No	4659 (91.6)	2654 (57.0)	2005 (43.0)	
Yes	427 (8.4)	244 (57.1)	183 (42.9)	
Diarrhea				**0.01**
No	4762 (93.6)	2734 (57.4)	2028 (42.6)	
Yes	325 (6.4)	165 (50.8)	160 (49.2)	
Fever				**<0.001**
No	4199 (82.5)	2450 (58.3)	1749 (41.7)	
Yes	888 (17.5)	449 (50.6)	439 (49.4)	
Illness with a cough				0.70
No	4276 (84.1)	2431 (56.9)	1845 (43.1)	
Yes	811 (15.9)	467 (57.6)	344 (42.4)	

**Table 2:** Bivariate analysis of childhood anemia according to the household- and community-level factors.

Variables	Total weighted (%)	Non-anemia, n (%)	Anemia, n (%)	p-value
**Household-level**				
Sex of household head				0.05
Female	492 (9.7)	260 (52.8)	232 (47.2)	
Male	4596 (90.3)	2639 (57.4)	1957 (42.6)	
Age of household head, years				**0.003**
≤ 24	219 (4.3)	114 (52.1)	105 (47.9)	
25–34	1434 (28.2)	864 (60.3)	570 (39.7)	
35–44	1162 (22.8)	678 (58.3)	484 (41.7)	
> 44	2271 (44.7)	1242 (54.7)	1029 (45.3)	
Ethnics				**<0.001**
Lao-Tai	2818 (55.4)	1536 (54.5)	1282 (45.5)	
Mon-Khmer	1338 (26.3)	726 (54.3)	612 (45.7)	
Hmong-Mien	737 (14.5)	521 (70.7)	216 (29.3)	
Chinese-Tibetan	145 (2.9)	87 (60.0)	58 (40.0)	
Other	48 (0.9)	28 (58.3)	20 (41.7)	
Education of household head				**0.02**
None	805 (15.8)	425 (52.8)	380 (47.2)	
Primary	2203 (43.3)	1270 (57.6)	933 (42.4)	
Secondary	1349 (26.5)	764 (56.6)	585 (43.4)	
Post-secondary	729 (14.3)	439 (60.2)	290 (39.8)	
Education of mother				0.19
None	1141 (22.4)	669 (58.6)	472 (41.4)	
Primary	2049 (40.3)	1184 (57.8)	865 (42.2)	
Secondary	1383 (27.2)	759 (54.9)	624 (45.1)	
Post-secondary	514 (10.1)	286 (55.6)	228 (44.4)	
Household wealth				0.48
Poorest	1331 (26.2)	774 (58.2)	557 (41.8)	
Poorer	1072 (21.1)	623 (58.1)	449 (41.9)	
Middle	1002 (19.7)	561 (56.0)	441 (44.0)	
Richer	905 (17.8)	496 (54.8)	409 (45.2)	
Richest	777 (15.3)	445 (57.3)	332 (42.7)	
Size of family				0.55
<4	454 (8.9)	249 (54.8)	205 (45.2)	
4–6	2918 (57.4)	1685 (57.7)	1233 (42.3)	
7–9	1275 (25.1)	722 (56.6)	553 (43.4)	
> 9	441 (8.7)	244 (55.3)	197 (44.7)	
Source of drinking water				0.25
Improved	4187 (82.3)	2401 (57.3)	1786 (42.7)	
Unimproved	901 (17.7)	498 (55.3)	403 (44.7)	
Type of toilet facility				0.30
Improved	3563 (70.0)	2047 (57.5)	1516 (42.5)	
Unimproved	1524 (30.0)	852 (55.9)	672 (44.1)	
Mosquito bed net				**0.01**
No	281 (5.5)	180 (64.1)	101 (35.9)	
Yes	4086 (94.5)	2719 (56.6)	2087 (43.4)	
**Community-level**				
Area of residence				0.06
Urban	1305 (25.7)	751 (57.5)	554 (42.5)	
Rural with road	3170 (62.3)	1775 (56.0)	1395 (44.0)	
Rural without road	612 (12.0)	373 (60.9)	239 (39.1)	
Region of residence				**<0.001**
North provinces	1588 (31.2)	1003 (63.2)	585 (36.8)	
Central provinces	2393 (47.0)	1291 (53.9)	1102 (46.1)	
South provinces	1107 (21.8)	605 (54.7)	502 (45.3)	

### Prevalence of anemia

The mean Hb level in the study population was 11.09 ± 1.28 g/dL. The overall prevalence of anemia among children aged 6–59 months was 43.0% (95% CI, 41.6%–44.3%). The prevalence of anemia was significantly higher among patients aged 6–11 months (72.6%) who had diarrhea (49.2%), and fever (49.4%) in the last 2 weeks prior to the survey in comparison to an older aged patients who had non-diarrhea and non-fever (Table 1). Moreover, the prevalence of anemia was higher among children who had a female household head (47.2%), a younger household head (47.9%), and an uneducated household head (47.2%) and those lived in the central region (46.1%) in comparison to children who had a male household head, an older household head, educated household head and those lived in the northern region (Table 2).

### Factors associated with childhood anemia using multilevel analysis

This study identified various factors associated with anemia among children aged 6–59 months in the Lao PDR. After controlling for covariates, multilevel logistic regression analysis showed that at the individual-level, male (AOR, 1.16; 95% CI, 1.01–1.34) and underweight children (AOR, 1.30; 95% CI, 1.09–1.55) were more likely to be anemic than female and non-underweight children (Table 3). However, children aged 12–23 months (AOR, 0.50; 95% CI, 0.38–0.65), 24–35 months (AOR, 0.18; 95% CI, 0.14–0.23), 36–47 months (AOR, 0.15; 95% CI, 0.11–0.20) and 48–59 months (AOR, 0.12; 95% CI, 0.09–0.16) were less likely to be anemic than those aged 6–11 months.

**Table 3:** Association and variation between individual-, household-, and community-level factors and childhood anemia.

Variable	Crude OR (95% CI)	p-value	Adjusted OR (95% CI)	p-value
**Individual-level**				
Sex of child				
Female	1		1	
Male	1.19 (1.04–1.36)	**0.008**	1.16 (1.01–1.34)	**0.01**
Age of child				
6–11 months	1		1	
12–23 months	0.50 (0.38–0.65)	**<0.001**	0.50 (0.38–0.65)	**<0.001**
24–35 months	0.18 (0.14–0.24)	**<0.001**	0.18 (0.14–0.23)	**<0.001**
36–47 months	0.16 (0.12–0.21)	**<0.001**	0.15 (0.11–0.20)	**<0.001**
48–59 months	0.12 (0.09–0.16)	**<0.001**	0.12 (0.09–0.16)	**<0.001**
Underweight				
No	1		1	
Yes	1.09 (0.93–1.28)	0.26	1.30 (1.09–1.55)	**0.003**
Diarrhea				
No	1		1	
Yes	1.29 (0.98–1.70)	0.06	1.00 (0.74–1.36)	0.96
Fever				
No	1		1	
Yes	1.41 (1.18–1.68)	**<0.001**	1.19 (0.98–1.45)	0.06
**Household-level**				
Sex of the household head				
Female	1		1	
Male	0.84 (0.69–1.07)	0.16	1.05 (0.81–1.37)	0.68
Age of household head				
≤ 24	1		1	
25–34	0.69 (0.48–0.98)	**0.04**	0.74 (0.50–1.09)	0.13
35–44	0.71 (0.50–1.02)	**0.06**	0.71 (0.48–1.06)	0.10
> 44	0.87 (0.61–1.22)	0.42	0.77 (0.52–1.13)	0.18
Ethnics				
Lao-Tai	1		1	
Mon-Khmer	1.11 (0.94–1.33)	0.20	1.23 (0.99–1.53)	0.05
Hmong-Mien	0.46 (0.37–0.59)	**<0.001**	0.45 (0.34–0.60)	**<0.001**
Chinese-Tibetan	0.74 (0.51–1.07)	0.11	0.90 (0.58–1.40)	0.65
Other	0.90 (0.44–1.84)	0.78	0.86 (0.40–1.84)	0.69
Education of household head				
None	1		1	
Primary	0.78 (0.64–0.96)	**0.02**	0.74 (0.58–0.94)	**0.01**
Secondary	0.80 (0.64–1.00)	0.05	0.79 (0.60–1.04)	0.09
Post-secondary	0.69 (0.54–0.90)	**0.006**	0.58 (0.42–0.81)	**0.001**
Education of mother				
None	1		1	
Primary	1.02 (0.85–1.23)	0.78	0.93 (0.75–1.16)	0.54
Secondary	1.19 (0.97–1.45)	0.09	1.05 (0.82–1.35)	0.67
Post-secondary	1.19 (0.91–1.55)	0.18	1.20 (0.85–1.69)	0.29
Mosquito bed net				
No	1			
Yes	0.72 (0.53–1.97)	**0.03**	0.79 (0.56–1.11)	0.18
**Community-level**				
Area of residence				
Urban	1		1	
Rural with road	1.07 (0.90–1.28)	0.42	1.07 (0.87–1.33)	0.48
Rural without road	0.90 (0.68–1.17)	0.44	0.97 (0.70–1.33)	0.85
Region of residence				
Northern provinces	1		1	
Central provinces	1.51 (1.27–1.79)	**<0.001**	1.59 (1.30–1.95)	**<0.001**
Southern provinces	1.58 (1.27–1.95)	**<0.001**	1.42 (1.11–1.81)	**0.005**
**Random effect variance**				
Community-level			0.24 (0.13–0.44)	0.001
Household-level			1.05 (0.85–1.30)	<0.001
**Model fit statistics**				
(DIC) -2 log-likelihood			22706.85	

At the household level, many factors were inversely associated with anemia, including education level of the household head (primary level [AOR, 0.74; 95% CI, 0.58–0.94] and post-secondary level [AOR, 0.58; 95% CI, 0.42–0.81] and Hmong–Mien ethnicity (AOR, 0.45; 95% CI, 0.34–0.60).

At the community level, the odds of developing anemia increased among children living in central provinces (AOR, 1.59; 95% CI, 1.30–1.95) or southern provinces (AOR, 1.42; 95% CI, 1.11–1.81) compared to that those children living in the northern provinces.

## Discussion

To the best of our knowledge, this is the first study to estimate the factors associated with anemia among children aged 6–59 months using multilevel analysis, which was conducted using a nationally representative sample of children aged <5 years. In this nationally representative survey of 5,087 children aged 6–59 months in the Lao PDR, 43.0% children had of anemia. The prevalence of anemia has slightly increased from the previous national prevalence in 2006 (40.9%) [13] and 2011 (42.0%) [1]. Anemia has remained a severe public health problem based on the WHO classification [3]. This study suggests that the Lao PDR has not achieved the national nutrition strategy, which is aimed at reducing the prevalence of childhood anemia by 30% and 20% in 2015 and 2020, respectively [21]. Although the Lao PDR has a comprehensive national nutrition policy, strategy, and plan of faction since 2008 [22], the implementation and enforcement of these policies are lacking. In particular, the lack of nationwide interventions to address the problem of anemia among Lao children is a major public health challenge.

In this study, after controlling for individual-, household-, and community-level factors, several factors were associated with an increase and decrease odds of developing childhood anemia. At the individual level, our study found an increased odds of developing anemia among male than female children. Previous studies in India [23], Brazil [24], Guinea-Bissau [12] and sub-Saharan Africa [25] have confirmed this association. This may be explained by the physiological variations, due to longitudinal and faster growth rates in boys than in girls [26–28], which requires a higher demand for iron for their bodies [23, 24], particularly during the early years of life [29]. In addition, the odds of developing anemia were reduced with the increasing age of children (12–59 months) compared to the first years of life (6–11 months) in our study. This association is consistent with the findings of a study in the Lao PDR [14] and other studies in different settings [10, 30]. A possible explanation is based on the fact that the concentration of iron and other micronutrients in human milk is relatively low [31, 32]. Inadequate iron intake [33] contributes to the development of anemia among infants aged 12 months after exclusive breastfeeding for 6 months [34]. Additionally, inadequate intake of appropriate diverse food is an important factor that could contribute to the onset of anemia at the early age in children because sticky rice, which is a staple food in the Lao PDR, has low iron content, ranging from 0.6–1.0 mg per 100 g of rice [35]. It is usually given to infants as major complementary food [36]. In addition, we found that underweight children had an increased odds of developing anemia. Malnutrition is a major public health challenge in the Lao PDR. A recent study reported a high prevalence of underweight (50.3%) among children aged <5 years in rural communities [37]. Underweight is associated with acute and chronic malnutrition, which share common causes with anemia [8], and these factors are aggravated by food insecurity and poverty [5]. Food insecurity and inadequate consumption of micronutrients such as iron, vitamin B12, and folate affect the nutritional status of children, which contributes to the development of anemia [6, 8]. Therefore, public health interventions should be targeted at improving the nutritional status of children by introducing diverse complementary foods enriched with micronutrients such as iron, vitamin B12, and folate from infancy.

At the household level, there was a reduced odds of developing anemia among children of Hmong–Mien ethnicity. Geographic differences may be responsible for this association because as the Hb level increases with the increasing altitude, particularly at altitudes >1,000 m [38]. The Hmong–Mien ethnic population mostly resides in mountainous areas in the northern region of the Lao PDR, where the average altitudes are 1,500 m above sea level. A previous study in Peru found a higher prevalence of anemia among children living in low-altitude areas than in those living in high-attitude areas [39]. Surprisingly, our study found that children of household having heads with primary and post-secondary education levels were less likely to be anemic in comparison to those children of household having heads with uneducated. Although, the majority of children’s caretakers were female, the households were predominantly headed by a male, who had higher educational levels [40] with better chances of having better-paid jobs and income, and therefore, greater access to iron-rich food for the family, including children. Additionally, wage disparities are relatively high between sexes in the Lao PDR [41].

At the community level, we demonstrated that children living in central or southern provinces were more likely to be anemic than those living in the northern provinces of the country. The central or southern provinces are considered to be higher malaria-endemic regions than the northern provinces [42]. A previous study conducted among children in a southern province of the country reported that children with positive asymptomatic malaria were five times more likely to be anemic than those with no malaria [15]. Moreover, the prevalence of known soil-transmitted helminths, such as hookworm, *Ascaris lumbricoides*, *and Trichuris trichiura*, which cause anemia among children [43, 44], was higher prevalence among preschool-age children in the southern province than in the northern province, especially the prevalence of hookworms (71.2% vs. 5.4%) [45, 46]. Furthermore, the prevalence of underweight was particularly high among children living in the southern region of the country, which could potentially contribute to childhood anemia among children living in this region [47].

### Limitations

The LSIS II is a nationally representative survey and provides a large health database that could be used to determine factors associated with anemia among children aged 6–59 months throughout the country. However, this study has some limitations. First, the cross-sectional study design does not make it possible to establish a cause–effect relationship. Second, several known risk factors for anemia, including parasitic infections such as malaria, and dietary information were not including in this study. Third, due to a lack of data on the altitudes of areas of residence of the participants, it is plausible that anemia in these children could have been overestimated.

## Conclusions

To the best of our knowledge, this is the first study to use a large sample of children aged 6–59 months in the Lao PDR to identify factors associated with anemia using multilevel analysis. The prevalence of anemia among children in the Lao PDR is a major public health concern. In this multilevel analysis, we found that childhood anemia was associated with various factors, including sex, age, underweight, ethnicity, educational level of the household head, and region of residence. These factors were identified as having significant random effects at the community and household levels. Therefore, to resolve the problem of anemia in the Lao PDR, an effective approach is needed to address each factor associated with childhood anemia. Interventions should consider three factors—male sex, underweight, and region of residence—that increase the odds of developing anemia. Moreover, to improve the health and well-being of anemic children aged <5 years in the Lao PDR, it is necessary to focus strongly on improving the nutritional status of the children, and prevention of childhood anemia should be considered as a major priority of public health interventions.

## Supporting information

S1 Fig Flowchart of sample selection. (PDF)
