# Supplementary figures and images for: Prevalence of anemia and its associated factors among children aged 6–59 months in the Lao People’s Democratic Republic: A multilevel analysis
Source: PLoS One. 2021 Mar 25;16(3):e0248969. doi: 10.1371/journal.pone.0248969 (PMC7993607; doi:10.1371/journal.pone.0248969)

**S1 Fig. Flowchart of sample selection**

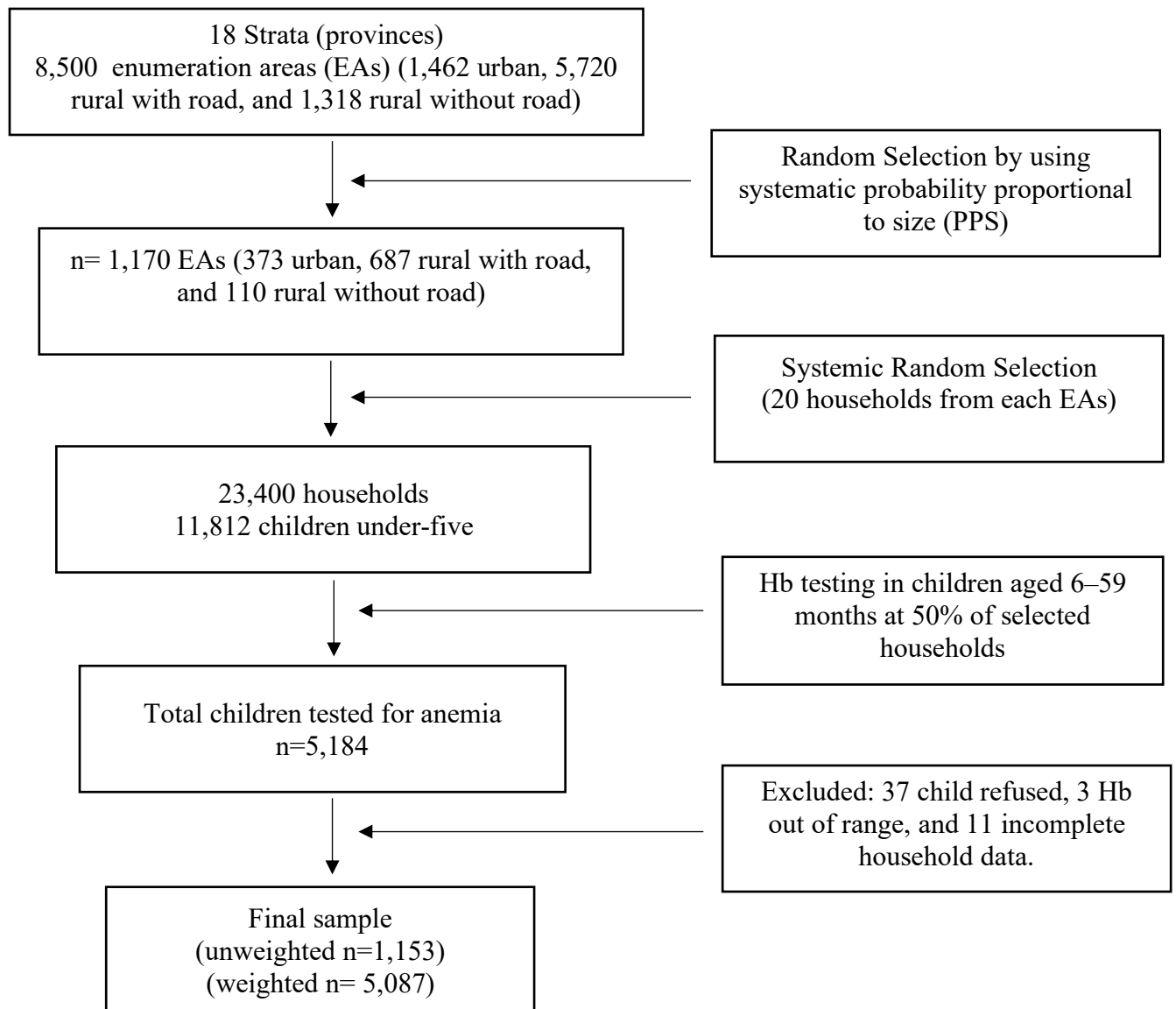

Supplement: S1 Fig — (PDF) [file pone.0248969.s001.pdf]
